# Detecting cathepsin activity in human osteoarthritis via activity-based probes

**DOI:** 10.1186/s13075-015-0586-5

**Published:** 2015-03-20

**Authors:** Louisa Ben-Aderet, Emmanuelle Merquiol, Duha Fahham, Ashok Kumar, Eli Reich, Yael Ben-Nun, Leonid Kandel, Amir Haze, Meir Liebergall, Marta K Kosińska, Juergen Steinmeyer, Boris Turk, Galia Blum, Mona Dvir-Ginzberg

**Affiliations:** Laboratory of Cartilage Biology, Institute of Dental Sciences, Hebrew University of Jerusalem, PO BOX 12272, Ein Kerem Campus, Jerusalem, 9112001 Israel; School of Pharmacy- Institute for Drug Research, Hebrew University of Jerusalem, PO BOX 12065, Ein Kerem Campus, Jerusalem, 9112001 Israel; Joint Replacement and Reconstructive Surgery Unit, Orthopaedic Surgery Complex, Hadassah Mount Scopus Hospital, Jerusalem, Israel; Department of Orthopaedics, Laboratory for Experimental Orthopaedics, Justus-Liebig-University of Giessen, Giessen, Germany; Department of Biochemistry and Molecular and Structural Biology, Jozef Stefan Institute, Ljubljana, Slovenia

## Abstract

**Introduction:**

Lysosomal cathepsins have been reported to contribute to Osteoarthritis (OA) pathophysiology due to their increase in pro-inflammatory conditions. Given the causal role of cathepsins in OA, monitoring their specific activity could provide means for assessing OA severity. To this end, we herein sought to assess a cathepsin activity-based probe (ABP), GB123, *in vitro* and *in vivo*.

**Methods:**

Protein levels and activity of cathepsins B and S were monitored by immunoblot analysis and GB123 labeling in cultured primary chondrocytes and conditioned media, following stimuli with tumor necrosis factor alpha (TNFα) and/or Interleukin 1 beta (IL-1β). Similarly, cathepsin activity was examined in sections of intact cartilage (IC) and degraded cartilage (DC) regions of OA. Finally, synovial fluid (SF) and serum from donors with no signs of diseases, early OA, late OA and rheumatoid arthritis (RA) patients were analyzed with GB123 to detect distinct activity levels of cathepsin B and S.

**Results:**

Cathepsin activity in cell lysates, conditioned media explants and DC sections showed enhanced enzymatic activity of cathepsins B and S. Further histological analysis revealed that cathepsin activity was found higher in superficial zones of DC than in IC. Examining serum and SF revealed that cathepsin B is significantly elevated with OA severity in serum and SF, yet levels of cathepsin S are more correlated with synovitis and RA.

**Conclusions:**

Based on our data, cathepsin activity monitored by ABPs correlated well with OA severity and joint inflammation, directing towards a novel etiological target for OA, which possesses significant translational potential in developing means for non-invasive detection of early signs of OA.

**Electronic supplementary material:**

The online version of this article (doi:10.1186/s13075-015-0586-5) contains supplementary material, which is available to authorized users.

## Introduction

Osteoarthritis (OA) is an extremely common type of degenerative joint disease and is a major cause of pain and chronic disability in aging individuals. Articular cartilage (AC) inflammation has often been attributed to OA development as a secondary effect, because increased matrix degradation and cell death occur under these conditions [1-6]. Specifically, synovial TNFα and IL-1β have been shown to drive matrix breakdown through their ability to upregulate cartilage catabolic enzymes as matrix metalloproteinases (MMPs) and downregulate anabolic matrix components [2,4-6]. To date, AC loss or damage in OA is detected on radiography by measuring decreased joint space width. However, radiographic evidence is seen only after significant cartilage degradation has already taken place. Hence early stages of OA may remain latent and asymptomatic for many years. Therefore, there is a pressing need for reliable new biomarkers and diagnostic tests that can facilitate earlier diagnosis of OA, and inform the prognosis, monitoring and therapeutic strategies for chronic and disabling forms of the disease [7].

Of particular interest is the family of cysteine proteases, cathepsins, which have been implicated in the pathogenesis of OA and contribute to cartilage degradation [8-13]. Extensive studies in mice, guinea pig, canine and rabbit models have shown a chondroprotective effect of cathepsin K inhibitors on OA pathology [14-16]. Cathepsin K has been shown to disrupt collagen type I and II fibrillar structure by cleaving their triple helices and altering the capacity of the fibers to withstand tensile forces [17]. Cathepsin K has thus been associated with OA, rheumatoid arthritis (RA) and osteoporosis pathogenesis [8]. Specifically, cathepsin K is actively secreted by resorbing osteoclasts and therefore, also impacts the gross density of bone. The fact that cathepsin K could be involved in many prominent musculoskeletal diseases such as OA, RA and osteoporosis [8,14-16] insinuates that it may not be a distinct etiological component in OA pathophysiology.

While other cartilage matrix degrading enzymes such as MMPs and A Disintegrin And Metalloproteinase with Thrombospondin Motifs (ADAMTs) are active at neutral pH, most of the lysosomal cathepsins are optimally active at acidic pH. Nonetheless, pH levels have been reported to drop in RA and OA superficial cartilage zones and synovial fluid (SF) [9,18]. Both cathepsin B and S have been reported to maintain their enzymatic activity in a wide pH range (from 5.0 to 7.5), [12,19-21], and therefore are likely to participate in cartilage extracellular matrix (ECM) degradation [22]. Specifically, cathepsin B was found to generate the CTX-II from the C-telopeptide of collagen type II and cleave aggrecan between the G1 and G2 domains [23], while cathepsin S was reported to display aggrecanase activity [24], suggesting they both have a role in accelerated cartilage degradation [24,25]. In addition to its extracellular targets in cartilage, cathepsin B was found to mediate cleavage and inactivation of SIRT1 (FLSIRT1) in response to pro-inflammatory stimuli, thereby leading to reduced *COL2A1* expression [12,26]. Furthermore, cathepsin B has displayed enhanced activity in OA versus non-OA/normal cartilage and was thereby envisioned to perpetuate OA pathophysiology in numerous investigations [10-12,27,28]. On the other hand, cathepsin S was recently shown to be secreted by chondrocytes stimulated with TNFα/IL1β [9], further supporting its role in joint inflammation.

**Figure 1:**
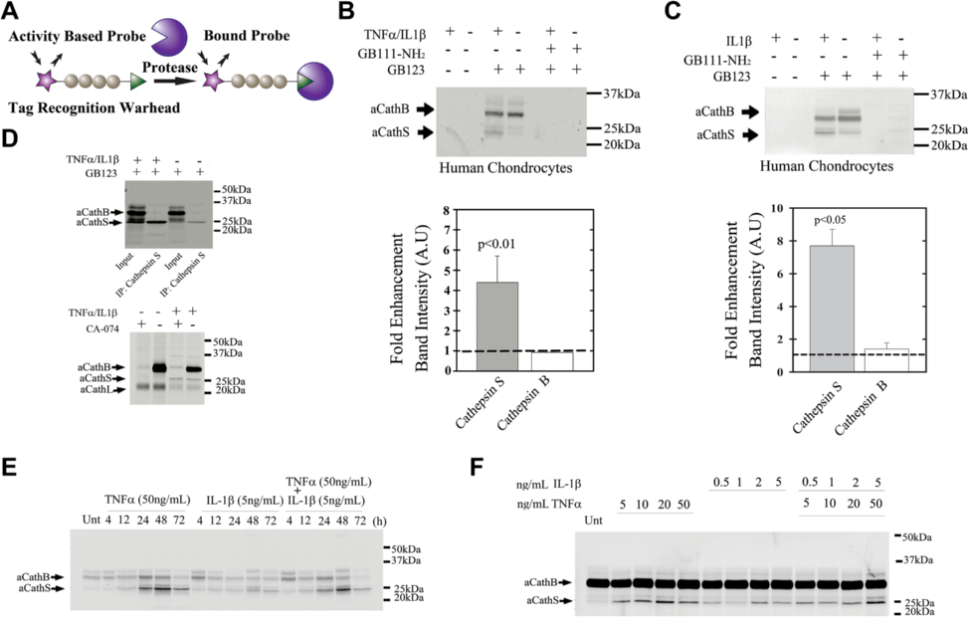
**Activity-based probe (ABP) targets active cathepsins in TNF** **α/IL1** **β-stimulated chondrocytes.** **(A)** ABP binding mechanism. Illustration shows the general structure of an APB with a triangular reactive moiety. Covalent modification of the target protease results in production of a fluorescently labeled enzyme. **(B)** Protein extracts from TNFα/IL1β-treated and untreated chondrocytes labeled with GB123 with or without pretreatment of cathepsin inhibitor GB111-NH_2_, as indicated (n = 4) with quantification of band intensity (Image J software) below. **(C)** Protein extracts from IL1β-treated and untreated chondrocytes labeled with GB123 with or without pretreatment of cathepsin inhibitor GB111-NH_2_, (n = 4) with quantification below. **(D)** Protein extracts from TNFα/IL1β-treated and untreated chondrocytes were labeled with GB123 and immunoprecipitated with cathepsin S antibody (n = 4). Below, a gel showing protein extracts incubated with or without CA-074 (selective cathepsin B inhibitor) and labeled with GB123. The long chain of active Cathepsin L was detected between 20 and 25 kDa. **(E)** Time-course experiments (50 ng/mL TNFα and 5 ng/mL IL1β) with GB123 labeling; experiment duration indicated above the gel (n = 4). **(F)** Dose response experiments (24-h incubation), with GB123 labeling, performed for cytokine concentrations (ng/mL);(n = 4). Gels were scanned (Typhoon scanner Excitation/Emission 630/670 nm) to detect fluorescent-labeled active cathepsin bands. aCathB, active cathepsin B (32 kDa); aCathS, active cathepsin S (25 kDa); IP, immunoprecipitation, Unt, untreated. Band intensity plots (image J software) show SD around average data point. Statistical analysis was by non-parametric Mann-Whitney analysis, considering *P* <0.05 statistically significant.

This research investigation sought to profile cathepsin B and S activity in primary chondrocytes, explant cultures, and serum and SF samples to examine if changes in cathepsin activity correlate with OA severity. To profile changes in cathepsin B and S activity we utilized a small molecule activity-based probe (ABP) that covalently binds to its target enzyme in an activity-dependent manner (see Figure 1A). The covalent nature of binding of ABPs to their enzyme targets leads to their retention in the active site allowing for biochemical analysis of each of their target enzymes simultaneously. ABPs can be attached to various tags such as fluorescent tags, biotin or radioactive markers that enable specific detection of their target proteases. Here we utilize our published fluorescent ABPs GB123 comprised of an electrophilic acyloxymethyl ketone warhead that generates a specific covalent modification to the active site cysteine of cathepsins B, L and S. GB123 can freely penetrate cells thereby targeting a larger pool of active intracellular and extracellular enzymes resulting in high signals *in vivo* detected non-invasively [29,30]. Furthermore, we have published on cathepsin ABPs tagged with ^64^Copper that can be suited for detection in humans [31]. Hence, using such probes provides translational potential in non-invasive imaging of the joints as well as bodily fluids.

## Materials and methods

### Human tissues

All procedures were performed with Hadassah Medical Center Institutional Review Board approval and in accordance with the Helsinki Declaration of ethical principles for medical research involving human subjects. Formal written informed consent was obtained from 30 donors with OA, who were undergoing total knee arthroplasty (mean age 72 years, mean body mass index (BMI) 31.5 kg/m^2^), prior to obtaining articular cartilage samples from their knee joints. Age-matched non-OA cartilage was obtained from NDRI, Philadelphia.

Serum and synovial fluid were obtained from non-OA donors and patients with early OA, late OA and RA, as partly described previously [32], following institutional approval by the local ethics committee of Justus-Liebig-University of Giessen, and obtaining written informed consent from all participants.

SF was obtained postmortem from the knee joints of 11 donors with no documented history of joint disease. The average (av) age of the non-OA donors was 22.5 years and the av BMI was 22.6. The control subjects were examined at the Institute of Forensic Medicine, Justus-Liebig-University of Giessen. Serum samples were obtained from 10 healthy donors (av age 22.5 years, BMI 22.6, 40% male).

SF (total of 32 samples) and serum (total of 20 samples) were ranked for OA severity based on the macroscopic appearance of six cartilage surfaces [33], namely the patella, trochlea, and medial and lateral femur and tibia, using the Outerbridge scale [34]. Joints with an av Outerbridge score ≤2 were classified as having early OA, and joints with an av Outerbridge score of ≥2 were classified as having late OA [33]. We collected serum from early OA (10 patients, av age 47.3 years, av BMI 27.6, 70% male), SF from early OA (17 patients, av age 44.8 years, av BMI 26.8, 75% male), serum from late OA (10 patients, av age 68.3 years, av BMI 28.2, 60% male), SF from late OA (15 patients, av age 67 years, av BMI 29.4, 69% male). SF was also collected from RA patients (10 patients, av age 64.6 years, av BMI 27.3, av serum C-reactive protein (CRP) 25.9 mg/L, 70% male) who were diagnosed by a board-certified rheumatologist, according to the American College of Rheumatology (ACR) 1987 revised criteria [34].

Serum samples were filtered with a 50-kDa cutoff amicon filter (Millipore, Billerica, MA, USA) and then labeled with our published cathepsin activity-based probe, GB123 (specified below) at 2 μM concentration for 3 hours. Reaction was stopped with sample buffer and 24-μg proteins were analyzed by SDS PAGE. Synovial fluids, 10 μL, were labeled with 5 μM GB123 for 3 hours, and then the reaction was stopped with sample buffer and analyzed by fluorescent SDS PAGE scanning using a Typhoon flatbed scanner (GE Healthcare: Bio-Sciences AB, Uppsala, Sweden, Excitation/Emission 630/670 nm). GB123 fluorescently labeled cathepsins B, L and S bands represent enzyme activity in proportion to band intensity, not enzyme quantity, as described previously [30].

### Cells, tissue cultures and reagents

Freshly isolated chondrocytes were obtained from intact and degenerative regions of OA-derived cartilage, dissected and subjected to 0.25% Trypsin-EDTA solution (Beit-Haemek Kibutz, Israel) at 37°C, 60 minutes rotation, then washed with PBS twice and overnight digested with 0.02% collagenase (Worthington Biochemical, Lakewood, NJ, USA) in DMEM containing 10% FCS and 1% Penicillin-streptomycin (Biological Industries, Beit-Haemek Kibutz, Israel). On the following day the cells were filtered through 40-μm nylon mesh, washed twice with PBS and lysed in radioimmunoprecipitation assay (RIPA) buffer (PBS, 1% NP-40, 0.5% Deoxycholate (DOC), 0.1% SDS). Proteins were quantified using the Bradford assay and 50 μg of proteins from each sample were mixed 1:1 with acetate buffer pH 5.5 (10 mM MgCl_2,_ 8 mM DTT, 100 mM Acetate) and with 1 μM GB123 (final concentration) for 1 hour at 37°C for cathepsins labeling. Intact cartilage (IC) and degraded cartilage (DC) supernatants were treated with collagenase and directly labeled with 1 μM GB123 for 5 hours at 37°C. Supernatants were filtered using a 50-kDa cutoff amicon filter (Millipore, MA), proteins were precipitated with 70% acetone at −80°C. On the following day the samples were centrifuged for 15 minutes at 14,000 rpm, pellets were resuspended in 35 μl RIPA buffer, and 30 μg of proteins were separated by SDS-PAGE.

To test if cathepsin S or B were modified in the presence of collagenase digestion media, 0.5 μg recombinant human cathepsin B or S [35], were incubated (1h or overnight) with collagenase media at 37°C and analyzed for cathepsin activity and protein levels. GB123 potency towards human cathepsin B, K and S was determined by labeling 0.5 μM active recombinant enzyme with 1 μM GB123 for 1 hour as indicated above.

Human chondrocytes were isolated from intact cartilage zones as indicated above and cultured to reach 90% confluence (passage 0 or 1) in DMEM media containing 10% FCS, 1% Penicillin-streptomycin at 37°C, and 5% CO_2_. Medium was then replaced with BIO-MPM (Biological Industries), a defined and enriched growth medium containing Penicillin-streptomycin with or without 50 ng/mL TNFα and/or 5 ng/mL IL1β (PeproTech, Rockey Hill, NJ, USA) for 24 hours. Cells were pretreated with a specific cathepsin inhibitor GB111-NH_2_ [36] or vehicle for one hour prior to direct labeling with 5 μM GB123. Then the cells were lysed in RIPA buffer and medium proteins were precipitated with 70% acetone as indicated above. Samples were analyzed by SDS PAGE or immunoprecipitated (IP) with cathepsin S antibody (R&D Systems, Minneapolis, MN, USA, cat#AF1183). To examine cathepsin B labeling (that is, 32 kDa), cells were lysed in RIPA and an equal amount of proteins were pre-incubated for 1 hour with 5 μM CA-074 (Sigma Aldrich, St Louis, MO, USA), a specific cathepsin B inhibitor, or DMSO vehicle at 37°C. Then 1 μM GB123 was added for an additional hour at 37°C. The reaction was stopped by adding sample buffer, followed by SDS PAGE. All samples were separated on a 12.5% SDS PAGE gel and scanned for fluorescence using a Typhoon flatbed scanner.

Three-dimensional alginate cultures were obtained with passage-1 human chondrocytes, treated with cytokines and processed for mRNA isolation, as previously described [37]. Explants cultures were obtained from intact or degenerated regions of cartilage derived from OA patients and dissected perpendicular to subchondral bone into round 4-mm-diameter cartilage tissue samples (weight 6 to 9 mg each) and analyzed for GB123 fluorescence.

### Immunoblot analysis

Cell pellets were suspended with RIPA lysis buffer with complete inhibitor cocktail (Roche, Basel, Switzerland), quantified with Bradford reagent (Sigma-Aldrich, St Louis, MO, USA), run on standard 10% SDS PAGE gels and transferred to polyvinylidene fluoride (PVDF) membranes for immunoblot (IB). The following antibodies were used for IB: β-actin (Santa Cruz Biotechnology, Dallas, Texas, USA; cat#47778), cathepsin S (Abcam, Cambridge, UK cat#134157), cathepsin B (Abcam cat# ab58802); Secondary antibodies: Anti-Mouse-alkaline-phosphatase (AP)-conjugated (Sigma-Aldrich, cat#, A3562), Anti-rabbit-AP conjugated (Sigma-Aldrich, cat#, A3687). All IBs were scanned at high resolution and band intensity was determined using Image J software (NIH, USA). After subtracting the background, each band was normalized to the corresponding housekeeping protein (that is, β-actin) appearing on the blot. Band intensity was presented in arbitrary units (A.U.) adjacent to the representative IB.

### Fluorescent labeling of explant sections

Explants were embedded in optical cutting temperature (OCT) and snap-frozen in liquid nitrogen prior to cryosection; and 10-μm sections were obtained and embedded on glass slides, fixed with 4% paraformaldehyde (diluted with PBS) for 30 minutes at 4°C, followed by washing three times with PBS for 5 minutes. Sections were labeled with GB123 (1 μΜ, 37°C, 30 minutes) with or without pre-incubation with GB111-NH_2_ inhibitor (1 μΜ, 37°C, 30 minutes). Slides were washed twice with PBS and mounted with 4′: 6-diamidino-2-phenylindole (DAPI)-fluoromount-G (SouthernBiotech, Birmingham, AL, USA). The stained sections were visualized under an inverted fluorescent microscope with DAPI and Cy5 lasers and filters (Olympus FV10i inverted microscope, Tokyo, Japan).

### Immunohistochemistry

IC/DC tissues were fixed in 4% formalin and decalcified for 3 days in 4% formic acid/4% HCl and embedded in paraffin: 5-μm sections were digested with 1 mg/mL Hyaluronidase (Sigma Aldrich, St Louis, MO, USA) in PBS (pH = 6) for 1 hour at 37°C, and stained with anti-cathepsin S antibody and DAB substrate kit (Zytomed systems, Berlin, Germany).

### Quantitative polymerase chain reaction (qPCR) analysis

mRNA was isolated using RNeasy mRNA purification columns (Qiagen, Hilden, Germany). cDNA was then prepared using the OneStep RT-PCR kit according to the manufacturers guidelines (Invitrogen Carlsbad, CA, USA). Real-time PCR reactions were performed using an ABI qPCR model 7300 or 7900 (Applied Biosystems, Foster City, CA, USA) with purified samples containing a Syber Green mix (Applied Biosystems) in accordance to the manufacturers’ guidelines. Primers for quantitative PCR were prepared for the following human genes: *Cathepsin S(CAT)*, forward: 5′-GACTGGAGAGAGAAAGGGTGTGTT-3′, reverse: 5′-CAGCTTTCCTGTTTTCAGCTTCA-3′, *hβ2MG*-F: ACCCCCACTGAAAAAGATGAG; reverse: ATCTTCAAACCTCCATGATGC; *GAPDH*-F: TACTAGCGGTTTTACGGGCG; R: TCGAACAGGAGCAGAGAGCGA.

### Statistical analysis

All experiments were performed on multiple donor samples as indicated in each figure legend. Data points represent average and standard deviation, unless otherwise indicated. Statistical analysis was obtained using non-parametric Mann-Whitney analysis (two-tailed). *P*-values for plotted data ≤0.05 were considered statistically significant. Correlation between serum and SF cathepsin bands (imageJ, A.U.) were drawn for age per clinical group (that is, healthy/non-OA, early OA, late OA and RA) and amongst all groups, based on the Pearson coefficient (1 ≥ p ≥ −1), considering *P* ≤0.05 to be statistically significant. The values were also assessed by linear regression (*R*^2^).

## Results

### Detection of enhanced cathepsin S activity in TNFα/IL1β-treated chondrocytes

We set out to investigate the cathepsin activity in OA by applying our previously developed fluorescently labeled cathepsin ABP, GB123, [30]. This ABP was designed to covalently attach to active cathepsins B (32 kDa), L (approximately 22 kDa) and S (25 kDa) (see illustration in Figure 1A) [36]. Surprisingly, we found that GB123 can label cathepsin K (25 kDa) as well, but significantly weaker compared to cathepsins B and S (Additional file 1).

GB123 was pre-incubated with chondrocyte extracts, which were stimulated or unstimulated with TNFα and IL1β. A more than four-fold increase in cathepsin S activity was detected (25-kDa band), without detectable changes in cathepsin B activity (Figure 1B). While cathepsin S activity was significantly enhanced, cathepsin B did not show a significant increase upon IL1β stimuli alone (Figure 1C). To identify the GB123-labeled bands we immunoprecipitated the labeled lysates with a cathepsin S antibody, and similarly, the pulled-down protein showed an increase in activity with TNFα/IL1β treatment (Figure 1D). Although the 25-kDa band may contain active cathepsin K the IP experiment confirmed the elevation in cathepsin S activity. To examine the exact band of cathepsin B, we subjected cell lysates to a specific cathepsin B inhibitor (CA-074) and labeled them with GB123 (Figure 1D, lower panel). The results confirm that the 32 kDa band represents active cathepsin B, given that it disappears when treated with the inhibitor without impacting the activity of cathepsin S (25 kDa). Overall, while no dramatic changes in chondrocyte cathepsin B activity (32 kDa) were observed, enhanced cathepsin S activity was clearly seen in TNFα- and/or IL1β-stimulated chondrocytes. These data are in line with time course and dose response experiments (Figure 1E and F, respectively), showing that cathepsin S is responsive to increasing cytokine dose. As cathepsin L activity (the band between the 20- to 25-kDa protein ladder) was variable, we chose to focus on cathepsins B and S, which exhibited consistent responsiveness to stimuli.

### Enhanced secretion of cathepsins B and S in conditioned media of cytokine-treated chondrocyte cultures

**Figure 2:**
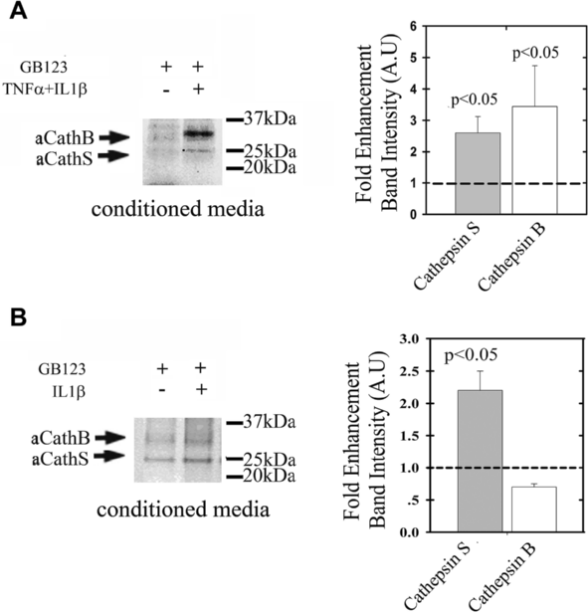
**Cathepsin activity in conditioned media of human chondrocytes and explant cultures.** Human chondrocytes were isolated from intact regions of osteoarthritis (OA)-derived cartilage and plated at passage 0. Following confluence the cells were treated with **(A)** 50 ng/mL TNFα and 5 ng/mL IL1β for 24 h or **(B)** 5 ng/mL IL1β. Conditioned media were labeled with GB123, concentrated and run on PAGE, and labeled cathepsins were detected by on gel-fluorescent scan (n = 4). Plots of band intensity (imageJ software) show standard deviation surrounding an average data point. Statistical analysis was obtained using non-parametric Mann-Whitney analysis, assuming *P* <0.05 to be statistically significant.

Conditioned medium from TNFα/IL1β-treated chondrocytes was collected and labeled with GB123. Enhanced cathepsin B and S activity was detected in TNFα/IL1β-conditioned medium (Figure 2A), with a similar trend for cathepsin S upon IL1β stimuli (Figure 2B). These data support the idea that pro-inflammatory stimuli contribute to catabolic enzyme secretion into the extracellular milieu in the cartilage.

### Enhanced cathepsin activity in degenerative cartilage

**Figure 3:**
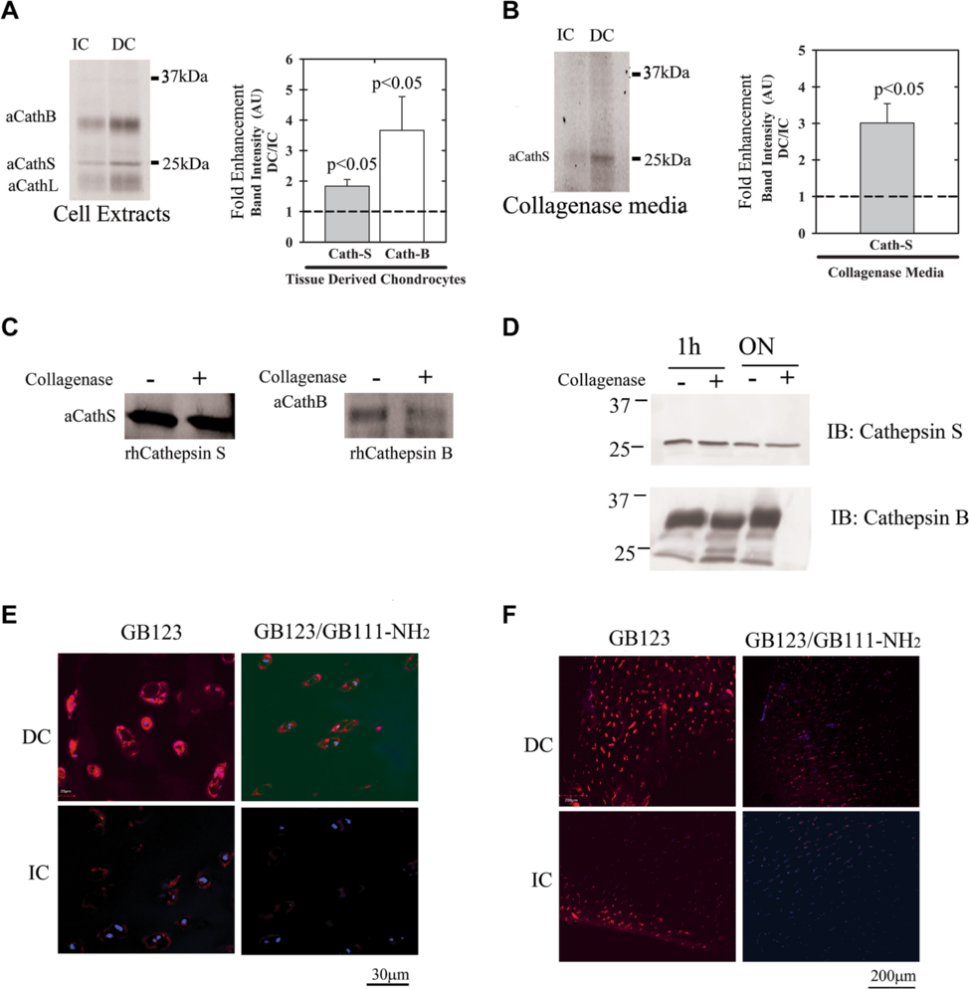
**Cathepsin activity is increased in degraded cartilage from osteoarthritis (OA) patients.** Intact and degraded cartilage (IC and DC) derived from end-stage OA patients were analyzed with GB123 for **(A)** cathepsin activity in freshly isolated chondrocyte lysates and **(B)** degraded cartilage media, which was obtained following a collagenase digestion procedure (24 h, 37°C). Samples were treated with GB123 and subjected to SDS PAGE and fluorescence visualization as in Figure 1 (n = 4). The heavy chain of active Cathepsin L was detected between 20 to 25 kDa **(A)**. **(C)** Recombinant cathepsin B (right) and cathepsin S (left) were treated for 24 h with collagenase digestion media (0.02%), labeled with GB123 and analyzed as in Figure 1 (n = 3). **(D)** Enzymes were analyzed by SDS-PAGE and immunoblotted with cathepsin B or S antibodies (n = 3). **(E,** **F)** Cryosections of IC/DC (5 μm-thick) stained with 4′: 6-diamidino-2-phenylindole (DAPI) (blue fluorescence for nuclei), GB123 (red fluorescence for cathepsin activity), with or without pre-incubation with the cathepsin inhibitor GB111-NH_2_ (n = 4). Increased cathepsin activity in DC versus IC regions is clearly observed. Images taken with 60-times magnification **(E)** and with 10-times magnification (n = 5) **(F)**. Plots of band intensity (panels **A** and **B**; imageJ software) show SD surrounding an average data point. Statistical analysis was by non-parametric Mann-Whitney analysis, assuming *P* <0.05 to be statistically significant.

To further investigate the cathepsin activity within different regions of OA cartilage, IC and DC regions were analyzed. The surrounding cartilage matrix was disintegrated by collagenase digestion and media and freshly isolated cells were subsequently labeled with GB123. While both cathepsins B and S showed enhanced activity in cell extracts from coarse DC regions (Figure 3A), only cathepsin S appeared enhanced (3-fold) in DC cartilage media (Figure 3B), as judged by its presence in collagenase digestion media. To examine whether treating cartilage tissue with collagenase modifies cathepsin activity, we incubated purified recombinant human cathepsins B and S separately with collagenase media (Figure 3C and D), as previously done with IC/DC samples. Only cathepsin B exhibited reduced activity when incubated with collagenase overnight (30% reduction in activity), while no changes were observed for cathepsin S activity (Figure 3C). These results fit well with the complete degradation of cathepsin B observed by IB after overnight incubation with collagenase, (Figure 3D). It is likely that the absence of active cathepsin B in DC collagenase digestion-media is due to its susceptibility to collagenase, which was significantly enriched in our digestion media.

Cryosections of IC and DC explants were stained with GB123 to visualize the regions with enhanced cathepsin activity. An inhibitor control (GB111-NH_2_) was used to verify the activity-dependent staining of the cathepsins (Figure 3E and F). Cells within DC regions showed markedly increased cathepsin activity compared to IC regions, observed by intense red fluorescence of the cells and the surrounding matrix.

**Figure 4:**
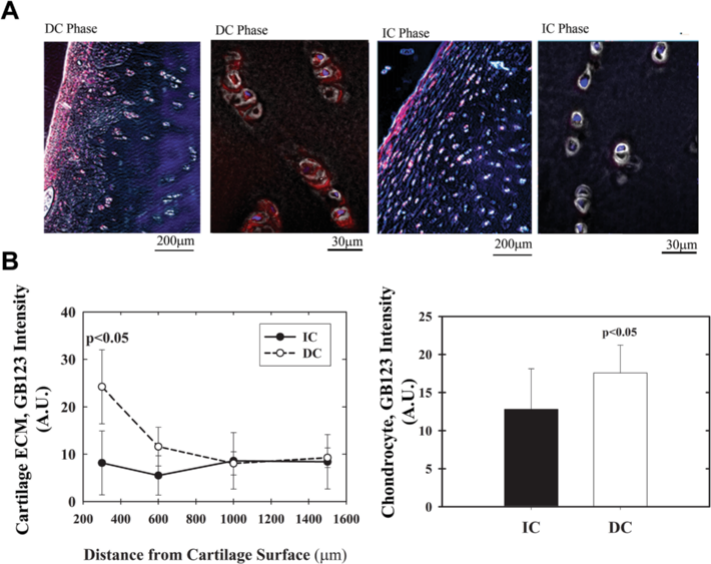
**Profiling cathepsin activity in osteoarthritis (OA) articular cartilage.** Cryosections of intact cartilage (IC)/degenerated cartilage (DC), 5-μm thick, stained with GB123 and 4′: 6-diamidino-2-phenylindole (DAPI) then analyzed for cathepsin activity using imageJ intensity software. **(A)** Representative phase images overlaid with GB123 fluorescence, sections of IC and DC at 60- and 10-times magnification (n = 5). Red and blue fluorescence are Cy5 (GB123) and DAPI, respectively. **(B)** Cathepsin activity as a function of cartilage distance from the articular surface (that is, superficial zone) and up to 1.5 mm deep (left panel): right panel, overall quantification of cathepsin activity surrounding chondrocyte clusters (that is, cartilage extracellular matrix (ECM)). Plots of band intensity (image-J software) show SD surrounding an average data point. Values were generated by subtracting the background from images of cathepsin-stained cell clusters. Statistical analysis was by non-parametric Mann-Whitney analysis, assuming *P* <0.05 to be statistically significant.

The exact location of the cathepsin activity was profiled within the superficial- and midzone-cartilage region samples of DC and IC stained with GB123 (Figure 4). Interestingly, articular cartilage ECM showed a significant (2-fold) increase in activity within the first 300-μm depth of DC samples, while this was not detected in IC (Figure 4B, left plot). Quantifying the fluorescence of cell clusters within superficial and midzone articular cartilage (that is, up to 1000 μm in depth) showed a 40% increase in cellular cathepsin activity within DC versus adjacent IC tissues, using GB123 labeling.

### Enhanced cathepsin S in pro-inflammatory conditions and during cartilage degeneration

**Figure 5:**
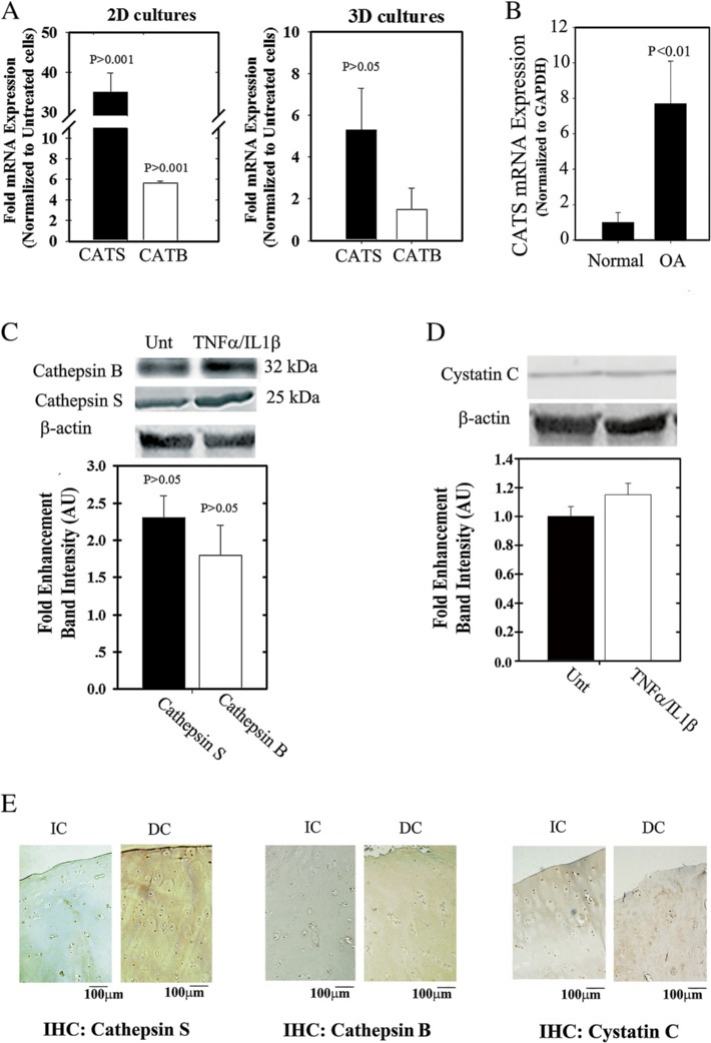
**Cathepsin S/B expression and activity is enhanced under cytokine stimulation.** **(A)** Fold-increase in cathepsin S and B (denoted as *CATS/B*) expression in cytokine (that is, TNFα and IL1β) stimulation of monolayer cultures (2D) and chondrocyte cultures in alginate microbeads (3D), normalized to GAPDH and untreated cells. For treated 3D cultures cytokines were added to the crosslinked alginate suspension containing chondrocytes. Unt indicates untreated controls. **(B)** Human chondrocytes were isolated from non-OA/normal and osteoarthritis (OA)-diagnosed donors, mRNA was isolated from samples and analyzed for *CATS* expression. **(C)** Protein extracts from human OA-derived chondrocytes (passage 0) that were treated or untreated with cytokines were immunoblotted for cathepsin S and B **(D)** and cystatin C. Band quantification plot beneath each blot in **(C)** and **(D)**. **(E)** Left panel shows immunohistochemistry (IHC) of IC/DC sections stained for cathepsin S, middle panel for cathepsin B and right panel for cystatin C (n = 3). Statistical analysis was by non-parametric Mann-Whitney analysis, assuming *P* <0.05 to be statistically significant.

Having shown that cathepsin B and S activities were enhanced (Figures 1 and 2), we next wanted to validate these findings also at mRNA level using monolayer and alginate microencapsulated human chondrocytes subjected to TNFα/IL1β stimulation (Figure 5A). We found that in chondrocytes stimulated with TNFα/IL1β expression of cathepsins S and B was enhanced (*CATS/B*), (Figure 5A; left panel). However, three-dimensional chondrocyte cultures showed mRNA increase only for *CATS* (Figure 5A; right panel). A significant increase in *CATS* expression was also observed in freshly isolated OA chondrocytes as compared to normal/non-OA chondrocytes (Figure 5B), which was similar to the significant increase in *CATB* expression that we previously reported [38]. In general, elevation in cathepsin S mRNA and protein was more pronounced than that observed under the same conditions for cathepsin B. In line with these results, the protein level of cathepsin S was also found elevated (>2-fold) in TNFα/IL1β chondrocytes, (Figure 5C), which is also apparent in immunohistochemistry of IC/DC sections (Figure 5E, left panel). Consistent with our previous reports [38], cathepsin B was also increased upon cytokine treatment (Figure 5C), but showed a less intense coloration within ECM of DC sections as compared to that observed for cathepsin S staining (Figure 5E, middle panel). The reduced coloration of cathepsin B in cartilage ECM may be due to its susceptibility to the presence of collagenase in OA tissue, as we previously showed in Figure 3. On the other hand, cystatin C (a natural cathepsin inhibitor) was not affected by cytokine stimuli of chondrocytes (Figure 5D); however, immunochemistry displayed more intense coloration within ECM of DC compared to IC sections (Figure 5E, right panel). Overall increased levels of cathepsin B/S compared to cystatin C may partly explain the reason for the enhanced cathepsin activity monitored with GB123 (Figure 4).

### SF and serum analysis for OA, RA and non-OA samples using GB123

**Figure 6:**
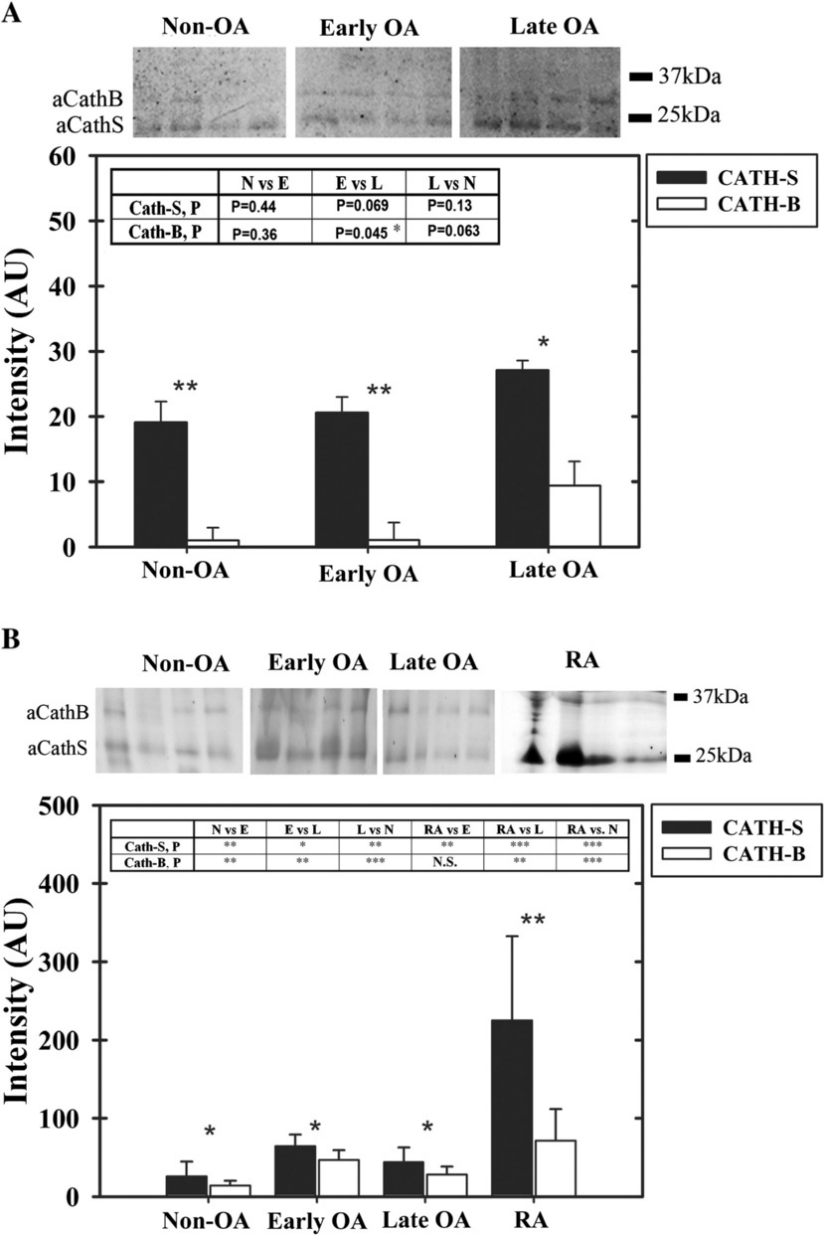
**Profiling cathepsin activity in serum and synovial fluid of osteoarthritis (OA) patients.** **(A)** Non-OA/normal (N), early OA (E) and late OA (L) sera were labeled with GB123, separated by SDS PAGE, scanned for fluorescence and quantified for band intensity, following subtracting gel background (n = 10 per group). **(B)** non-OA (N) (n = 11), early OA (E) (n = 17), late OA (L) (n = 15) and rheumatoid arthritis (RA) (n = 10) synovial fluid (SF) were labeled with GB123, and analyzed similar to **A**. Plots of band intensity (imageJ software) show SD surrounding an average data point. Statistical analysis was by non-parametric Mann-Whitney analysis, assuming *P* <0.05 to be statistically significant. Within each plot a table depicts statistical significance (*P <0.05, **P <0.01, ***P <0.001) for cathepsin B and S activity upon comparison of the different groups.

To test if cathepsin activity detected by ABP could serve as a marker for severity of OA and joint inflammation we analyzed serum samples of non-OA cadavers (N), and early OA (E) and late OA (L) patients, (Figure 6A). Using GB123 labeling we monitored cathepsin B and S bands, based on our *in*-*vitro* observations, their expected selectivity to the probe and characterized molecular weight. Furthermore, we confirmed that GB123 labels non-OA human SF in an activity-dependent manner, which was validated by pretreatment with a cathepsin inhibitor, GB111-NH_2_ (Additional file 2: Figure S2).

While cathepsin S is significantly more active in all OA sera than cathepsin B, only cathepsin B was significantly enhanced in sera of early OA versus late OA patients. Cathepsin B also showed a similar increase between non-OA and late OA sera, however, this increase was statistically insignificant. Given the activity of both cathepsins increased at late stages of OA, we next assessed their activity in SF from non-OA donors and patients with early OA, late OA and RA (Figure 6B). Among all the groups, cathepsin S activity was higher as compared to cathepsin B, similar to the trend observed in sera. Moreover, cathepsin S activity was substantially higher in RA as compared to early OA, late OA and non-OA samples. On the other hand, cathepsin B activity was enhanced with a higher statistical significance amongst the various OA stages than that of cathepsin S. It is therefore concluded that both cathepsins are equally important for diagnosing OA in SF. While cathepsin B activity may indicate the severity of OA, cathepsin S could exclude RA, since it is highly active in RA SF. With respect to OA disease severity, cathepsin B appears to exhibit delayed serum accumulation compared to equivalent levels in SF.

To assess if cathepsin activity is affected by age (Additional file 3), we carried out correlation assays for age versus cathepsin S/B intensity amongst all groups (in upper graphs for serum and SF) and within each group (B, Table, Additional file 3). The data suggest that age does not correlate significantly with changes in cathepsin S and B activity, based on *P*-values of the Pearson coefficient (*P*c). Nonetheless, analysis of cathepsin S activity in SF of RA diagnosed patients showed that it is significantly reduced with age, which coincides with the reduced cathepsin activity observed in advanced age reported by Bayliss *et al*., 1978 [28]. These data indicate that cathepsin S may be a good marker for non-invasive imaging of RA joints only at early ages (40 to 50 years), when OA signs due to cathepsin B are not evident (Figure 6B). Based on these preliminary findings, it appears that non-invasive joint imaging to detect cathepsin activity in SF may be a good indicator of early signs of OA.

## Discussion

The unique structure of cartilage ECM plays a key role in the load-bearing capacity of the AC. It is composed of collagen type II, IX and XI, which provide tensile force to the tissue [39]. In addition, proteoglycans, especially aggrecan, provide a hydrated gel-like medium enabling AC resilience to load-induced deformation. During OA, much of the cartilaginous ECM is degraded by catabolic enzymes namely ADAMTS’s and other MMPs [1,2,6]. Amongst the catabolic enzymes involved in cartilage breakdown, the lysosomal cysteine proteases, the cathepsins have also been shown to contribute to cartilage ECM degradation [8-11,22]. Interestingly, OA cartilage and SF have acidic pH [9,18], which may favor catabolic activity of lysosomal cysteine proteases.

Lysosomal cysteine proteases activity has been additionally linked to internal pathways of chondrocyte apoptosis and gene regulation related to OA pathology via regulation of SIRT1 activity [12,26,40,41]. Here we used an experimental approach wherein various chondrocyte cultures were stimulated with pro-inflammatory cytokines TNFα and IL-1β resulting in a prominent increase in cathepsin S and B activity. Among these cathepsins, cathepsin S was more susceptible to changes in cytokine stimulation and their doses and relatively resistant to collagenase breakdown, which may be enhanced in diseased cartilage. Despite the fact that the activities of both cathepsins were enhanced in DC chondrocytes, active cathepsin S showed a moderate increase in late OA serum with more abundant activity levels in RA SF. On the other hand, increased cathepsin B activity was observed in late OA serum and amongst the different OA severities within SF. Collectively, these data support the idea that cathepsin S is involved in acute inflammatory conditions of cartilage, in agreement with the earlier study of Požgan *et al*. (2012), [13]. In addition, previous reports by Hou *et al*. (2002) demonstrated that cathepsin S is pronounced in macrophage-like synoviocytes supporting the concept that it possesses a role in antigen presentation as well as matrix degradation of synovial tissues [42]. Cathepsin S also serves as a microglial mediator exerting increased pain sensitivity [43]. Clark *et al*., (2012) showed that inhibiting cathepsin S significantly reduces pain perception and attenuated microglial response in a collagen-induced arthritis rat model [44]. Given that the immune-related pathogenesis of RA involves pronounced synovial infiltration and synovitis, which is less pronounced in OA pathophysiology, cathepsin S activity may be less responsive than cathepsin B to the pathogenesis of OA, in line with our observations.

Superficial regions of severely degraded cartilage showed more than a 2-fold increase in cathepsin activity using ABP labeling. These data therefore support the idea that the cathepsins are involved in ECM processing and are regulated by factors in the SF [22]. Based on these observations and our analysis of SF we envision that imaging techniques using modified ABPs for better joint penetration may be suitable means for early detection of OA, rather than serum analysis with these probes. Furthermore, fluorescently labeled ABPs were already converted to ^64^Copper labeled cathepsin positron emission tomography (PET) probe [31] to improve detection in deeper tissues. Such means could provide additional insight into the joint state using non-invasive imaging techniques based on PET. Upon systemic administration, ABPs are envisioned to access the synovial space through its vasculature, potentially providing a signal-to-background ratio indicative of the cathepsin activity in the joints, which could be translated to OA severity within the joint.

There have been recent reports on molecular imaging of diseased joint with fluorescently labeled small molecules in an attempt to image OA [45-47]. These reports prove that imaging reagents may enter and accumulate in the diseased joint and could also be monitored non-invasively in mice. Thus, our ABP could potentially accumulate in OA joints when applied *in vivo*. We find the ABPs attractive imaging reagents because they covalently bind their target *in vivo*, thereby accumulating in the diseased area, such as SF, and allow for non-invasive imaging [28]. Moreover, the cathepsin ABPs enable quantifiable biochemical analysis of their targets, enabling careful monitoring of the various cathepsins *in vivo*.

## Conclusion

In summary, our analysis of SF using ABP show that cathepsin B is more likely involved in OA pathogenesis than cathepsin S and that their differential detection using ABP labeling could provide a measureable diagnostic tool for detecting RA or early stages of OA. Overall, based on our results, we envision that development of robust and sensitive imaging procedures based on ABPs will enable early detection of OA and RA as well as provide means for testing the efficacy of disease-modifying drugs to treat joint diseases.

## Additional files

Additional file 1:
**Recombinant human cathepsins (final 0.5 μM active enzyme per lane; reference [** 35 **]), were diluted with acetate buffer and labeled with 1 μM of GB123 for 1 hour at 37°C.** Reaction was stopped by adding sample buffer and boiling the samples at 95°C for 5 minutes. Samples were loaded on a 12.5% SDS PAGE gel and scanned with a Typhoon scanner as in Figure 1. Additional file 2:
**Osteoarthritis (OA) synovial fluid, 5µl, were diluted in acetate buffer 1:1 and passed through a syringe with 21-G, 27-G, and 30-G needles.** Samples were then filtered through a 50-kDa cutoff amicon and spun at 14,000 g at 18°C for 30 minutes. Samples were treated with dimethyl sulfoxide (DMSO) vehicle or 10 μM GB111-NH_2_ cathepsin inhibitor [36], for an hour at 37°C. After pretreatment, the samples were labeled with 5 μM GB123 for one hour at 37°C. Reaction was stopped by adding 4 × sample buffer and boiled for 10 minutes. Samples were separated on a 12.5% SDS PAGE and scanned for Cy5 fluorescence with a Typhoon scanner. Additional file 3:
**Correlation of age with cathepsin S (CTSS) and B (CTSB) activity on serum and synovial fluid (SF): serum and SF samples were processed and run as indicated in the Materials and methods section.** All gels were scanned in high resolution and band intensity was determined using ImageJ software. After subtracting the background, net band intensity (A.U.) corresponding to either cathepsin S or B was correlated with the donors age amongst all groups (A) and within each group (B, Table). Correlation between serum and SF cathepsin bands were based on Pearson coefficient test (Pc; 1 ≥ p ≥ −1), considering Pc ≤0.05 to be statistically significant. Pearson correlation coefficient measures linear correlation between two variables, wherein a *P*-value of 1 indicates total positive correlation, 0 indicates no correlation, and −1 is total negative correlation. The values were also assessed for linear regression (*R* ^2^), as indicated.

## Abbreviations

2D: monolayer chondrocyte cultures
3D: three-dimensional alginate chondrocyte cultures
ABP: activity-based probes
AC: articular cartilage
av: average
BMI: body mass index
CA-074: cathepsin B inhibitor, L-trans-epoxisuccinil-lle-Pro-OH propylamide
CTSB: cathepsin B
CTSS: cathepsin S
DAPI: 4′: 6-diamidino-2-phenylindole
DC: degenerated cartilage
DMEM: Dulbecco’s modified Eagle’s medium
DMSO: dimethyl sulfoxide
ECM: extracellular matrix
FCS: fetal calf serum
GB111-NH_2_: cathepsin inhibitor
HEPES: hydroxyethyl piperazineethanesulfonic acid
IB: immunoblot
IC: intact cartilage
IL1β: interleukin 1-beta
IP: immunoprecipitation
MMP: matrix metalloproteinase
mRNA: messenger RNA
OA: osteoarthritis
PBS: phosphate-buffered saline
PET: positron emission tomography
qPCR: quantitative reverse-transcriptase PCR
RA: rheumatoid arthritis
RIPA: radioimmunoprecipitation assay
SDS: sodium dodecyl sulfate
SF: synovial fluid
TNFα: tumor necrosis factor-α
