# Supplementary material for: Detecting cathepsin activity in human osteoarthritis via activity-based probes
Source: Arthritis Res Ther. 2015 Mar 20;17(1):69. doi: 10.1186/s13075-015-0586-5 (PMC4415352; doi:10.1186/s13075-015-0586-5)
Supplement: Additional file 1: — Recombinant human cathepsins (final 0.5 μM active enzyme per lane; reference [ 35 ]), were diluted with acetate buffer and labeled with 1 μM of GB123 for 1 hour at 37°C. Reaction was stopped by adding sample buffer and boiling the samples at 95°C for 5 minutes. Samples were loaded on a 12.5% SDS PAGE gel and scanned with a Typhoon scanner as in Figure 1. [file 13075_2015_586_MOESM1_ESM.pdf]

# Supplementary Data 1.

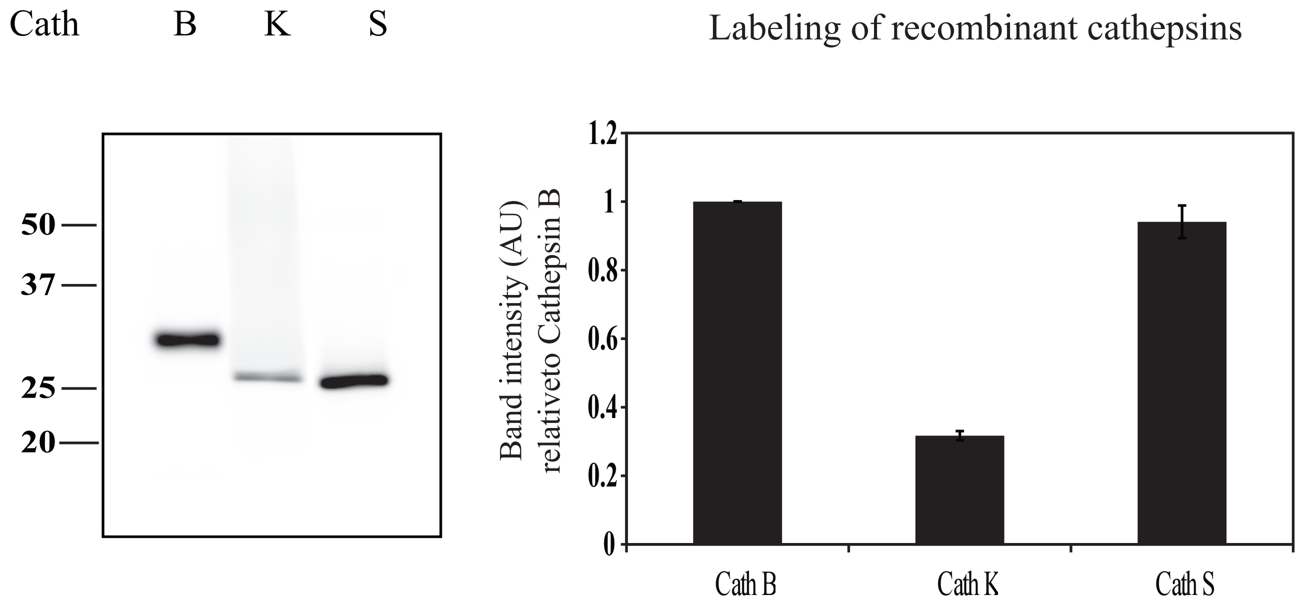


Supplementary data 1.
Recombinant human cathepsins (final 0.5$\mu$M active enzyme per lane; Ref 33), were diluted with acetate buffer and labeled with 1$\mu$M of GB123 for 1 hour at 37$^0$C. Reaction was stopped by adding sample buffer and boiling the samples at 95$^0$C for 5 min. Samples were loaded on a 12.5% SDS PAGE gel and scanned with a Typhoon scanner as in Figure 1.
