# Supplementary material for: Detecting cathepsin activity in human osteoarthritis via activity-based probes
Source: Arthritis Res Ther. 2015 Mar 20;17(1):69. doi: 10.1186/s13075-015-0586-5 (PMC4415352; doi:10.1186/s13075-015-0586-5)
Supplement: Additional file 2: — Osteoarthritis (OA) synovial fluid, 5µl, were diluted in acetate buffer 1:1 and passed through a syringe with 21-G, 27-G, and 30-G needles. Samples were then filtered through a 50-kDa cutoff amicon and spun at 14,000 g at 18°C for 30 minutes. Samples were treated with dimethyl sulfoxide (DMSO) vehicle or 10 μM GB111-NH2 cathepsin inhibitor [36], for an hour at 37°C. After pretreatment, the samples were labeled with 5 μM GB123 for one hour at 37°C. Reaction was stopped by adding 4 × sample buffer and boiled for 10 minutes. Samples were separated on a 12.5% SDS PAGE and scanned for Cy5 fluorescence with a Typhoon scanner. [file 13075_2015_586_MOESM2_ESM.pdf]

Supplementary Data 2.

Synovial Fluid labeled with GB123

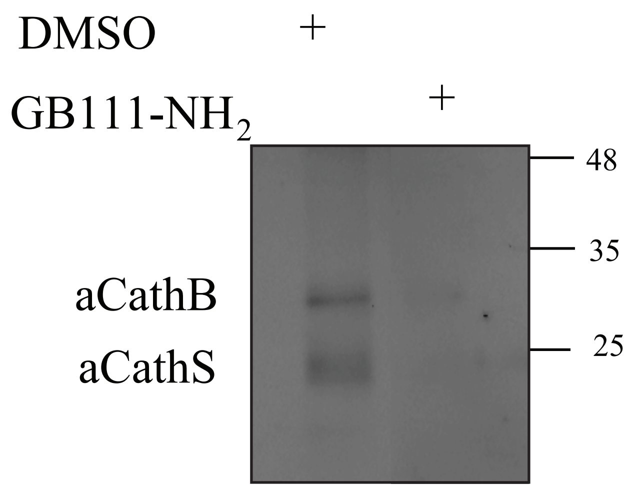

Supplementary Data 2.
Five μL of OA synovial fluid were diluted in acetate buffer 1:1 and passed throw a syringe with needles 21G, 27G, 30G. Samples were then filter through a 50kDa cutoff amicon and spun at 14000g at 18^{0}C for 30min. Samples were treated with DMSO vehicle or 10μM GB111-NH2 cathepsin inhibitor (34), for an hour at 37^{0}C. After pretreatment, the samples were labeled with 5 μM GB123 for one hour at 37^{0}C. Reaction was stopped by adding 4 x sample buffer and boiled for 10 min. Samples were separated on a 12.5 % SDS PAGE and scanned for Cy5 fluorescence with a Typhoon scanner.
