# Supplementary figures and images for: Detecting cathepsin activity in human osteoarthritis via activity-based probes
Source: Arthritis Res Ther. 2015 Mar 20;17(1):69. doi: 10.1186/s13075-015-0586-5 (PMC4415352; doi:10.1186/s13075-015-0586-5)

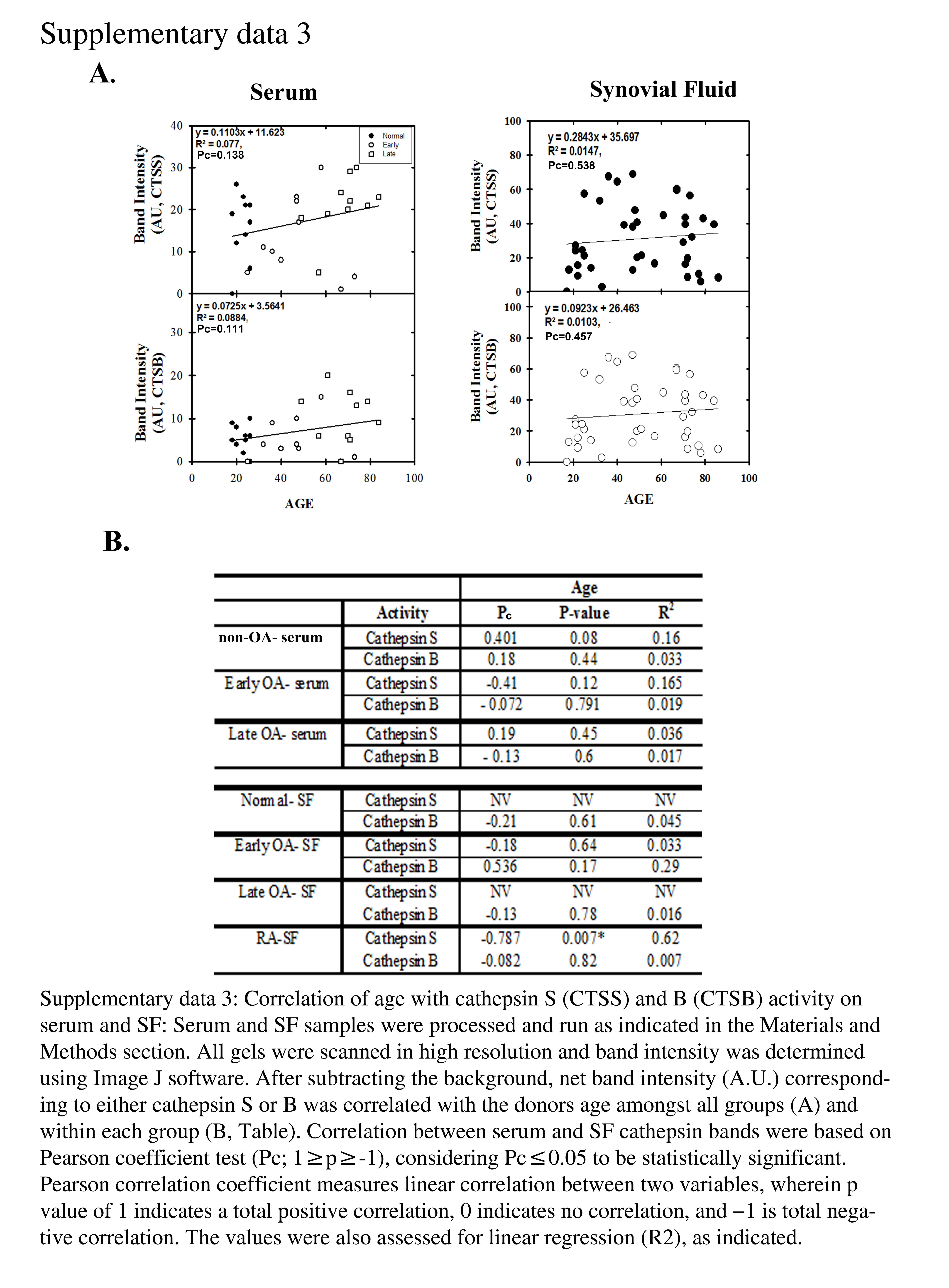

Supplement: Additional file 3: — Correlation of age with cathepsin S (CTSS) and B (CTSB) activity on serum and synovial fluid (SF): serum and SF samples were processed and run as indicated in the Materials and methods section. All gels were scanned in high resolution and band intensity was determined using ImageJ software. After subtracting the background, net band intensity (A.U.) corresponding to either cathepsin S or B was correlated with the donors age amongst all groups (A) and within each group (B, Table). Correlation between serum and SF cathepsin bands were based on Pearson coefficient test (Pc; 1 ≥ p ≥ −1), considering Pc ≤0.05 to be statistically significant. Pearson correlation coefficient measures linear correlation between two variables, wherein a P-value of 1 indicates total positive correlation, 0 indicates no correlation, and −1 is total negative correlation. The values were also assessed for linear regression (R 2), as indicated. [file 13075_2015_586_MOESM3_ESM.tiff]
